# A feasibility randomized controlled trial of ReaDySpeech for people with dysarthria after stroke

**DOI:** 10.1177/0269215517748453

**Published:** 2017-12-26

**Authors:** Claire Mitchell, Audrey Bowen, Sarah Tyson, Paul Conroy

**Affiliations:** 1 Division of Neuroscience and Experimental Psychology, University of Manchester, Manchester Academic Health Science Centre, Manchester, UK; 2 Central Manchester University Hospitals NHS Foundation Trust, Manchester Academic Health Science Centre, Manchester, UK; 3 Division of Nursing, Midwifery and Social Work, University of Manchester, Manchester Academic Health Science Centre, Manchester, UK

**Keywords:** Dysarthria, stroke, randomized controlled trial, rehabilitation interventions

## Abstract

**Objective:**

To evaluate the feasibility of a multicentre randomized controlled trial of ReaDySpeech, an online speech therapy programme for people with dysarthria.

**Design:**

Feasibility randomized controlled trial, 2:1 minimization procedure.

**Setting:**

Four UK NHS services across hospital and community.

**Participants:**

Forty participants with dysarthria at least one week post-stroke.

**Interventions/comparator:**

ReaDySpeech with usual care (*n* = 26) versus usual care only (*n* = 14).

**Main outcomes:**

Feasibility measures included the following: recruitment and retention rate, time taken to carry out assessments, success of outcome assessor blinding, fidelity and adherence. Participant baseline and outcome measures collected before and after 8–10 weeks of intervention were the Frenchay Dysarthria Assessment II, Therapy Outcome Measure, Communication Outcomes After Stroke Scale, EQ-5D-5L and Dysarthria Impact Profile.

**Results:**

Recruited 40 participants out of 74 eligible people, 1–13 weeks post stroke and mean age 69 years (37–99). Retention was very high (92%). Assessor blinding was not achieved with intervention allocation correctly guessed for 70% of participants (26/37). Time to carry out assessments was acceptable to participants. ReaDySpeech was delivered to 16 of 26 allocated participants, who completed 55% of prescribed activities, but both interventions were delivered at low intensity (mean 6.6 face-to-face sessions of 40-minute duration).

**Conclusion:**

Recruitment and retention in this randomized controlled trial of computerized therapy for dysarthria is feasible for acute stroke. However, further feasibility work is needed to evaluate whether it is possible to recruit chronic stroke; increase intervention delivery, intensity and adherence; achieve outcome assessor blinding by video-recording and to determine sample size for a larger trial of effectiveness.

## Introduction

Dysarthria describes impaired speech intelligibility caused by weak, slow or uncoordinated muscles in the speech tract and is thought to affect 20%–30% of stroke survivors.^1,2^ This can be hugely disabling leading to social isolation and poor health outcomes.^3^ This disorder occurs when any of the respiratory, laryngeal and/or oral articulator muscles; tongue; lips; cheeks; and palate are affected.^4^ Severity of symptoms may range from being completely unintelligible, to slow speech and articulation difficulties, or intelligible speech where the speaker works hard through internal rehearsal and monitoring to ensure their speech output is of good quality.^5^

Intervention for dysarthria typically involves specific exercises, advice, explanation, strategies or psychological support depending on the individual’s needs and goals. Impairment level intervention such as breathing exercises to improve breath support or control as well as non-speech, oro-motor movements trying to improve strength, speed or accuracy of oral muscle movement may be used. Activity-level strategies to improve intelligibility such as slowing speech down, over-articulating words, text-to-speech aids or alphabet charts to spell out letters or words may improve success of communication. Psychological support to support wider participation may include explanation and education about dysarthria, working with communication partners or communication support groups. Clinical need will often reflect the individual’s previous communication demands and their stage or acceptance of recovery.^5^

However, research is extremely limited and there is no robust evidence indicating which interventions work best, when treatment should start, nor optimal duration or intensity of treatment. The lack of adequately powered, well-controlled trials in dysarthria was illustrated in a recent Cochrane review.^6^ This is in marked contrast to the wealth of research on aphasia, the other main communication disorder experienced after stroke.^7,8^

Evidence from other areas of stroke rehabilitation indicates that high-intensity, repetitive task–specific practice may be the most effective way to promote motor recovery after stroke.^9^ Our overall aim was therefore to evaluate the effectiveness of interventions for dysarthria post-stroke. Anecdotally, patients with post-stroke dysarthria in clinical practice reported to the first author that paper-based dysarthria exercises were not particularly clear or motivating and asked whether there were computer-based alternatives. This led to the development of ReaDySpeech, an online programme, developed with clinicians and patients participating in one-to-one interviews and small group discussion. ReaDySpeech is a tailored programme where therapists select exercises and activities to improve intelligibility that is intended to be user-friendly, accessible and engaging with the expectation that this might increase uptake and, crucially, treatment intensity. Preliminary work, in accordance with Medical Research Council (MRC) guidance to develop a complex intervention,^10^ found ReaDySpeech to be acceptable to speech and language therapists and patients when used in routine clinical practice using a prospective observational and interview design^11^ which led to the feasibility trial reported here. The aim was to evaluate whether we could recruit and retain participants with a view to conducting a future large-scale trial of effectiveness comparing ReaDySpeech with usual care for people with dysarthria after stroke.

## Methods

We registered this trial with the International Standard Randomized Controlled Trials number register (ISRCTN84996500) and obtained ethical approval from the UK National Research Ethics Service Committee Northwest (15/NW/0371). Four patient research advisors formed the ‘Ever Ready’ group, advising on the design, conduct and dissemination of the trial.

Eligibility criteria were as follows: patients with post-stroke dysarthria: more than one week post stroke (no upper limit); medically stable: considered by their speech and language therapist as likely to benefit from intervention; and sufficient English language skills to participate in therapy without a translator. Patients with a co-occurring aphasia were eligible and were only excluded if the treating speech and language therapist felt that severity precluded the use of ReaDySpeech.

We recruited participants from four hospital and community-based stroke services in England over 14 months. The site speech and language therapists identified patients from their existing caseload or new referrals and recorded reasons for exclusion or declining participation. Those meeting the inclusion criteria who were interested in the study met the first author, who recorded informed written consent or reasons for declining study participation.

Exclusion criteria were as follows: co-existing progressive neurological conditions or cognitive hearing or visual problems that prevented use of ReaDySpeech as judged by the treating speech and language therapist who sought advice or further opinion from other health professionals if in doubt.

At baseline, before random allocation, we recorded the following: demographic data (age and gender), stroke information (time since stroke, type of stroke – haemorrhagic or infarction, and stroke classification), levels of pre-morbid and current functioning (modified Rankin scale^12^), current activities of daily living (Barthel Index^13^) and the co-existence of aphasia (severity, how it was diagnosed).

The following were completed at baseline and follow-up:

- Frenchay Dysarthria Assessment (second edition, FDA-II)^14^
- Dysarthria Therapy Outcome Measures Activity (TOMA)^15^
- Communication Outcomes After Stroke Scale (COAST)^16^
- Dysarthria Impact Profile (DIP)^17^
- EQ-5D-5L and visual analogue scale^18^

We explored the trial feasibility by monitoring recruitment and retention rates including reasons for declining participation and for withdrawal, time taken to carry out outcome assessments and the success or otherwise of outcome assessor blinding.

Data about how the intervention was delivered (face to face or independently) and by whom were extracted from therapists’ records. Exercise selection and completion data were captured by the ReaDySpeech software. Participants were also interviewed face to face by the first author about the impact the study set-up, assessments and interventions had on their involvement, using yes/no questions, open questions and 5-point rating scales about the trial and the intervention.

Following baseline measurements, participants were randomly allocated to ReaDySpeech with usual care or to usual care only. To ensure allocation concealment, a third-party system used minimization by the recruiting site and by time since stroke, acute (≤12 weeks post stroke) or chronic (≥12 weeks post stroke). Blinded outcome assessment by the first author using the measures listed above was carried out immediately after the 8–10 weeks intervention period.

### Interventions

In the control group, participants received usual care only which would vary by site, from no intervention to best practice guidelines.^19,20^ This could include the following: specific exercises for speech muscles, breathing, articulation work; strategies such as slowing speech, communication aids such as alphabet charts or text-to-talk aids, education about dysarthria and/or awareness training; and psychological support or advice and/or strategies to communication partners. Intervention details were recorded in speech therapy notes and retrieved following completion of follow-up.

In the intervention group, participants received usual care (as described above) and ReaDySpeech, an online computer programme, delivered in any way considered clinically appropriate by the treating therapist. ReaDySpeech was accessible using any Wi-Fi enabled device (smart phone, tablet or computer). ReaDySpeech included activities for articulation, breathing, rate of speech, volume, facial expression, intonation and oro-motor exercises.^19^ These activities were shown through video clips, instructions appearing on-screen and words, sentences or phrases appearing on-screen. Exercises were set by the therapist specific to each individual’s need and amended according to progress. Participants were able to choose which exercises to complete from the ones selected by the therapist and the programme automatically recorded both selection and completion. ReaDySpeech provided feedback on the completion of exercises but not on the quality of speech production. The ReaDySpeech programme could be used during face-to-face therapy sessions with a therapist initially and thereafter with an assistant, supported by family or independent practice. We wanted to explore how often the intervention was delivered and used. Although we expected duration to be up to 8 or 10 weeks, we did not specify the intensity or duration. Similarly, participants were able to practise independently if they wanted to but were not specifically required to do so. Both usual care and ReaDySpeech interventions are outlined in greater detail in the published protocol.^21^

### Data handling and analysis

The study was not designed to be statistically powered to test for a between-group difference in outcomes. Instead, we aimed to recruit 24 people to the ReaDySpeech intervention group and a minimum of 12 participants to the control group as recommended for a feasibility study.^22^

Recruitment and retention rates were analysed using descriptive summary statistics looking at patterns and reasons for non-participation. Outcome data were reported on all participants with an intention-to-treat approach^23^ from baseline to outcome using descriptive summary statistics: mean, standard deviation and 95% confidence intervals. We determined effectiveness of outcome assessor blinding by comparing guessed with actual allocation, presented as a percentage and analysed using the kappa statistic. We used exploratory analysis using summary statistics to report the feasibility of delivering the intervention by fidelity and adherence. Responses to interview questions were reported as similar themes but no formal qualitative analyses were used.

## Results

### Recruitment and retention of participants

**Figure 1.**
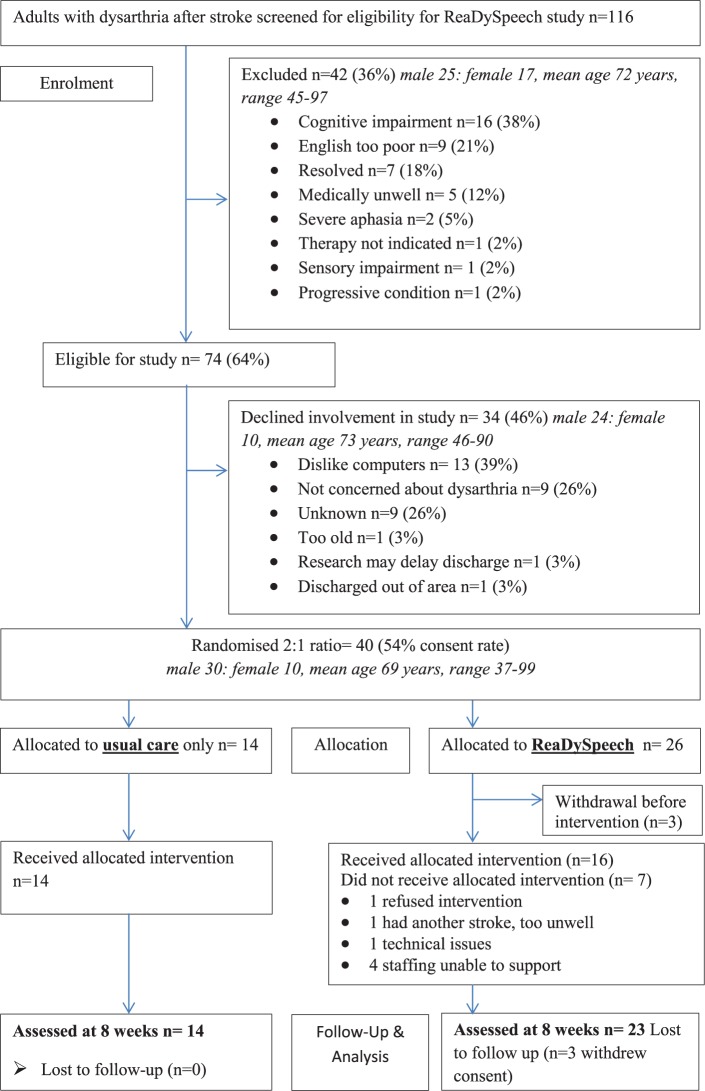
CONSORT flow diagram for participants.

We recruited 40 of the 74 eligible participants identified from the four participating sites between September 2015 and October 2016 (average rate 2.9/month over 14 months). Full details of recruitment and retention are shown as a CONSORT flow diagram^24^ in Figure 1, and participants’ baseline characteristics are described in Table 1.

**Table 1.** Baseline characteristics of participants by treatment allocation.

Characteristics	ReaDySpeech (*n* = 26)	Usual care (*n* = 14)
Mean age, years (min–max)	70 (37–99)	67 (55–85)
Male/female	18/8	12/2
Recruitment location		
Hospital	16	5
Community	10	9
Days post-stroke mean (min–max)	24 (8–67)	27 (8–90)
Aphasia present (severity)	2 (mild aphasia)	2 (mild aphasia)
Stroke severity, mean (standard deviation)		
Baseline Barthel Index, 0 dependent, 100 independent	56 (42.5)	83 (29.6)
Baseline Modified Rankin Scale, 0 no symptoms, 6 death	3 (1.4)	2 (1.5)
Stroke lesion location: lacunar, partial anterior circulation, total anterior circulation, posterior circulation	Lacunar = 11 Partial anterior circulation = 6 Total anterior circulation = 3 Posterior circulation = 4 Not known = 2	Lacunar = 7 Partial anterior circulation = 4 Total anterior circulation = 1 Posterior circulation = 1 Not known = 1

The most frequent reason for ineligibility was impaired cognition identified by the therapist. The main reason given by patients for declining involvement was a dislike of computers or low prioritization of dysarthria. There was no difference in age between those declining or participating. Co-existing aphasia was rarely reported as a reason for exclusion but only four recruits had aphasia (Table 1). Despite our open-ended eligibility criteria, we only recruited people with acute stroke as none of the patients in the participating sites were more than 13 weeks post stroke.

The study retention rate was high. In total, 37 out of the 40 recruits (8% attrition) completed all assessments. The interviews showed that participants understood the need for randomization and no-one withdrew because of their group allocation. The three participants lost to follow-up were all from the ReaDySpeech intervention group (Figure 1). They withdrew before they knew their intervention group but after randomization; one due to a misdiagnosis of stroke; one due to another health complication and one changed their mind due to other commitments. Having reviewed their reasons and the timing of withdrawal, we consider these three as missing completely at random and have not imputed outcome data.

### Feasibility of measurements

The groups (ReaDySpeech, *n* = 26; usual care, *n* = 14) were well matched at baseline for occurrence of aphasia (severity rated by therapist), lesion location and days post stroke (Table 1). However, the ReaDySpeech group had greater communication impairments and activity limitations, lower mood, quality of life and were more likely to be recruited in hospital.

We found it was feasible to carry out the Frenchay Dysarthria Assessment II,^14^ dysarthria activity level from the Therapy Outcome Measure,^15^ Communication Outcomes After Stroke Scale^16^ and EQ-5D-5L.^18^ However, it was not feasible to use the Dysarthria Impact Profile,^17^ which is designed for long-term adjustment to dysarthric symptoms, in this acute population. It took 60–90 minutes to complete all assessments at follow-up which was considered appropriate by 97% (36/37) of participants who felt these assessments reflected their speech and health difficulties.

The assessor blinding was unsuccessful. The assessor was un-blinded either explicitly or inadvertently in 11 cases (10 ReaDySpeech and 1 usual care). The assessor guessed treatment allocation correctly for 10 out of 14 in the usual care group and 16 out of 23 in the ReaDySpeech group. Observed agreement is 70% compared to 51% expected by chance. Kappa is 0.39 (*P* = 0.008) giving evidence of agreement (i.e. correct prediction) beyond chance.

Changes in outcome measures between baseline and follow-up are shown in Table 2. For the group as a whole, irrespective of allocation, outcomes improved over time. The standard deviation of the Frenchay Dysarthria Assessment II and Dysarthria Therapy Outcome Measure activity score at follow-up was considerably higher than that of its change score from baseline to follow-up, but this was not seen in the Communication Outcomes After Stroke Scale.

**Table 2.** Outcome measures from baseline to follow-up.

Outcome measure	Baseline, mean (standard deviation)			Outcome, mean (standard deviation)			Whole group, mean difference (SD, 95% CIs)
	All *n* = 40	UC *n* = 14	RS *n* = 26	All *n* = 37	UC *n* = 14	RS *n* = 23	
Impairment: FDA-II	All: 159 (37.5)	UC: 170 (20.2)	RS: 153 (43.3)	All: 179 (32.7)	UC: 184 (20.4)	RS: 177 (38.6)	21 (21.2, 27.8 to 14)
Activity: Dysarthria TOMs	All: 3 (1.0)	UC: 3.5 (0.8)	RS: 3.2 (1.1)	All: 3.7 (0.9)	UC: 3.9 (0.6)	RS: 3.6 (1.1)	0.5 (0.7, 0.7 to 0.2)
Participation: COAST	All: 59 (16.3)	UC: 63.1 (15.0)	RS: 56 (16.7)	All: 67 (16.1)	UC: 71 (15.3)	RS: 65.3 (16.6)	8.5 (16.6, 14 to 3)
Participant reported health quality of life states: EQ-5D-5L							
Visual analogue scale, median (IQR)	UC, *n* = 14, Median = 63 (25th = 50, 75th = 84)	RS, *n* = 26, Median = 50 (25th = 25, 75th = 64)		UC, *n* = 14, Median = 76.5 (25th = 55, 75th = 86)		RS, *n* = 23, Median = 65 (25th = 50, 75th = 80)	
EQ-5D-5L	Baseline problems (%)			Outcome problems (%)			
Mobility	UC 9 (64)	RS 24 (92)		UC 8 (57)		RS 16 (70)	
Self-care	UC 7 (50)	RS 21 (81)		UC 3 (21)		RS 15 (65)	
Usual activity	UC 8 (57)	RS 24 (92)		UC 7 (50)		RS 24 (92)	
Pain/discomfort	UC 6 (43)	RS 17 (65)		UC 7 (50)		RS 14 (61)	
Anxiety/depression	UC 3 (21)	RS 16 (62)		UC 4 (29)		RS 15 (65)	

UC, usual care; RS, ReaDySpeech; FDA-II, Frenchay Dysarthria Assessment II; Dysarthria TOMs, Dysarthria Therapy Outcome Measure activity score; COAST, Communication Outcomes After Stroke Scale.

### Feasibility of the intervention

In total, 16 of the 26 participants randomized to the ReaDySpeech group accessed the ReaDySpeech intervention. Of the 10 who did not, this was due to a lack of staffing for five participants (three from a single hospital); three withdrew before the intervention started (Figure 1). One refused intervention and one had another stroke but these two both agreed to follow-up.

For those receiving ReaDySpeech and usual care, face-to-face sessions were of similar low intensity (6.6 sessions per participant, min 1–max 24), with a mean session time of 43 minutes (SD 28: min 10–max 120 minutes). Different models of therapy provision were observed with assistants carrying out 81 of the 151 (54%) sessions in the ReaDySpeech group compared to 20 of the 95 (21%) sessions in usual care, and the rest were by qualified speech and language therapists. In the ReaDySpeech group, all of the face-to-face sessions used the programme, with exercise selection including impairment and activity-level exercises and two participants had psychological support.

For the 16 participants set up with access to ReaDySpeech, completion rate for the exercises was 55% across all sites. Of these 16 participants, nine used ReaDySpeech independently outside of treatment sessions (56%), mostly in the community with their own computers. Most found the programme straightforward and easy to use. They commented specifically on the videos, as well as being able to practise when it was convenient to them and reported an improved confidence in their speech. All would recommend ReaDySpeech to others.

## Discussion

This feasibility randomized controlled trial of ReaDySpeech for people with dysarthria post-stroke exceeded its recruitment target and found it is feasible to recruit and retain an acute population within the context of the NHS, provided staffing was in place. A lack of NHS therapy provision for people beyond the acute phase post stroke means people with chronic dysarthria were not easily identified. Further work will examine alternative routes to recruit people with chronic dysarthria, co-existing aphasia and those without computer confidence. Random allocation using minimization did not result in balance across groups on key variables that may be important for outcomes indicating the need for further minimization or baseline adjustment in a definitive trial. A future trial also needs to achieve assessor blinding (e.g. using videoed assessments) and to determine the sample size needed for adequate statistical power. ReaDySpeech delivery was difficult to achieve at sites with low-therapy staffing but the unexpectedly high rate of successful delivery at sites with assistants suggests a way forward. Intensity for both interventions was relatively low and although ReaDySpeech participants completed around half of the selected activities, further development of the technology should increase independent use.

Recruitment was carried out through initial identification by NHS speech and language therapists and proved feasible for recruiting early post-stroke. Recruitment to randomized controlled trials can be difficult, particularly for vulnerable participants early post-stroke,^25,26^ so we considered a recruitment rate of 54% to be reasonable. We targeted sites with acute and community care to recruit early and chronic stroke patients but found services did not deliver intervention beyond 12 weeks of stroke. It is important to find out what happens to these patients who remain concerned about their speech after early intervention but prioritize their communication later. We know nine of the eligible patients (26%) declined participation as speech was not an early priority. To widen recruitment to include chronic stroke, we will alter our recruitment strategy and target other providers of support and intervention such as charity, independent sector and online stroke forums.

Interestingly, we found that there were very few patients identified who also had a co-occurring aphasia and we recruited fewer (*n* = 4, 10%) than other studies (29%–31%^27,28^). A future trial could use clinical research practitioners to identify all stroke admissions with a communication impairment to reduce recruitment bias as well as therapist workload.^25,29,30^

The most likely reason for eligible participants to decline was due to a dislike of computers (*n* = 13, 38%) despite the treatment making minimal technical demands on users. Although technology is becoming ever more common, useful lessons have been learnt about how to describe the research and intervention more carefully.^31^ Describing tablets and smart phones instead of computers may be less daunting. Screening of a broader range of patients using carefully considered wording to describe the technology will be introduced in a future study.^25^

Retention rates were high in this trial with 37 out of 40 (92%) participants, followed up eight weeks after study entry. A future trial requires later follow-up to evaluate sustained improvement. Retention rates may be affected by later follow-up and other proposed changes such as recruiting a chronic stroke population and intensity of delivery and will be considered when calculating a future sample size.

Following randomized allocation by minimization, we found that the groups were not balanced at baseline on some measures. This could be dealt with by statistical adjustment when analysing a definitive trial or minimizing by severity of speech at the activity level, for example, the Dysarthria Therapy Outcome Measure activity score. The potential to use the latter as the primary outcome measure for a future trial will be discussed with patients when developing the protocol. When considering outcome measures for a future trial, it may be beneficial in terms of statistical power to assess the Frenchay Dysarthria Assessment II and Dysarthria Therapy Outcome Measure activity score at baseline to allow analysis of covariance due to the higher standard deviation at follow-up when compared to the change score from baseline to follow-up. Conversely, there was no suggestion of a similar reduction in standard deviation when comparing ‘follow-up’ with ‘change from baseline’ for the Communication Outcomes After Stroke Scale which suggests that there is no statistical benefit from baseline assessment of this scale with this population.

A key finding of the study was the unsuccessful assessor blinding of outcome measures as participants were often keen to discuss their intervention. This was particularly the case for those who had been allocated to ReaDySpeech, despite being asked not to disclose this. Video assessment, including the Therapy Outcome Measure, has been used to successfully blind the outcome assessor in other trials of communication impairment after stroke.^32,33^ Further feasibility work will explore whether the benefit of videos outweigh any adverse impact on recruitment and retention.

Frequency and intensity of ReaDySpeech and usual care delivery was low, particularly in acute hospital settings due to staffing levels and will be examined further alongside the need to ensure the programme meets the needs of all users. At two of the sites, therapists reported actively seeking out assistants to deliver the ReaDySpeech intervention and this warrants future health economic investigation. Just over half of the ReaDySpeech group used the online therapy programme for independent practice, and this study has raised awareness of some of the barriers to independent practice in acute settings. A future trial will be more prescriptive about how, when and how often to deliver it while considering the potential impact on adherence to treatment and retention. We will emphasize the philosophy of guided self-management underpinning ReaDySpeech through which intensity of engagement with the intervention could be achieved through flexible, self-administration by patients and whether this differs between acute and chronic populations. Independent use of ReaDySpeech was not readily facilitated in acute, in-patient settings (e.g. concerns about leaving tablets alone with patients) and further work will seek to avoid these barriers.

In conclusion, to ensure the success of a definitive trial, we will carry out a further feasibility trial around recruitment of chronic dysarthria, improvements to the programme for independent use, improving fidelity, adherence and intensity of ReaDySpeech, achieve assessor blinding by video-recording outcome assessments and to determine sample size.

**Clinical messages**

- People with dysarthria early after stroke are willing to engage in a randomized trial of ReaDySpeech, but alternative methods are required to access and recruit people with chronic dysarthria and it may be difficult to recruit sufficient numbers to a large-scale study.
- Blinding of outcome assessors was difficult to achieve with face-to-face outcome assessments.
